# *In vivo* imaging of hepatic neutrophil migration in severe alcoholic hepatitis with ^111^In-radiolabelled leucocytes

**DOI:** 10.1042/BSR20180466

**Published:** 2018-07-31

**Authors:** Jonathan R. Potts, Neda Farahi, Mark R. Howard, Mark R. Taylor, Sarah Heard, Arun N. Shankar, Graeme J. Alexander, Edwin R. Chilvers, Sumita Verma, A. Michael Peters

**Affiliations:** 1 Department of Gastroenterology and Hepatology, Brighton and Sussex University Hospitals NHS Trust, Brighton, U.K.; 2 Department of Medicine, Brighton and Sussex Medical School, Brighton, U.K.; 3 Department of Medicine, University of Cambridge School of Clinical Medicine, Cambridge, U.K.; 4 Department of Histopathology, Brighton and Sussex University Hospitals NHS Trust, Brighton, U.K.; 5 Department of Nuclear Medicine, Cambridge University Hospitals NHS Foundation Trust, Cambridge, U.K.; 6 Department of Hepatology, Cambridge University Hospitals NHS Foundation Trust, Cambridge, U.K.; 7 Division of Clinical and Laboratory Investigation, Brighton and Sussex Medical School, Brighton, U.K.

**Keywords:** alcohol-related liver disease, cirrhosis, inflammation, 111In-labelled leucocytes, liver biopsy, steatohepatitis

## Abstract

The study’s aim was to image severe alcoholic hepatitis (SAH) using ^111^In-labelled leucocytes with two objectives in mind: firstly for non-invasive diagnosis and secondly to provide a platform for experimental therapies aiming to inhibit intrahepatic neutrophil migration. ^111^In-leucocyte scintigraphy was performed 30 min and 24 h post-injection in 19 patients with SAH, 14 abstinent patients with alcohol-related cirrhosis and 11 normal controls. Eleven with SAH and seven with cirrhosis also had ^99m^Tc-nanocolloid scintigraphy. Change in hepatic ^111^In radioactivity was expressed as decay-corrected 24 h:30 min count ratio and, in SAH, compared with histological grading of steatohepatitis and expression of granulocyte marker, CD15. Hepatic microautoradiography on biopsy specimens obtained 24 h post-injection of ^111^In-leucocytes was performed in one patient. Median 24 h:30 min hepatic ^111^In activity ratio was higher in SAH (2.5 (interquartile range (IQR): 1.7–4.0) compared with cirrhotics and normal controls (1.0 (0.8–1.1) and 0.8 (0.7–0.9) respectively, *P*<0.0001). In SAH, it correlated with CD15 expression (r = 0.62, *P*=0.023) and was higher in marked compared with mild/moderate steatohepatitis (4.0 (3.0–4.6) compared with 1.8 (1.5–2.6), *P*=0.006). Hepatic-to-splenic ^99m^Tc count rate ratio was reduced in SAH (0.5 (0.4–1.4)) compared with cirrhotics (2.3( 0.6–3.0)) and three historic normal controls (4.2 (3.8–5.0); *P*=0.003), consistent with impaired hepatic reticuloendothelial function. Scintigraphic findings in SAH included prominent lung radioactivity at 30 min, likely the result of neutrophil primimg. Microautoradiography demonstrated cell-associated ^111^In in areas of parenchymal neutrophil infiltration. In conclusion, ^111^In-leucocyte scintigraphy can non-invasively diagnose SAH and could provide a platform for evaluation of novel treatments aiming to inhibit intrahepatic neutrophil migration.

## Introduction

Alcohol-related liver disease (ARLD) is a growing clinical problem. In the U.K., rates of hospitalisation and death have risen by almost 50% over the past decade, a trend which is projected to continue [1]. Acute alcoholic hepatitis (AH) is the most florid form of ARLD. It presents with jaundice and systemic upset in heavy drinkers. Clinically mild AH may resolve with abstinence but in-hospital mortality in severe AH (SAH), defined by a discriminant function (DF) ≥32, approaches 40% [2]. Prompt diagnosis is key to improving survival, enabling timely supportive care, nutritional support and potentially specific medical therapies such as corticosteroids (CS) [3]. Although histology is the gold standard for diagnosing SAH, liver biopsy is controversial because its accuracy varies from 50 to 90% [4–6]. In most instances, coagulopathy and ascites mandate transjugular biopsy (TJB), an invasive procedure with recognised morbidity and mortality [7]. Histological features include a neutrophil-rich parenchymal inflammatory infiltrate surrounding ballooned hepatocytes, often with eosinophilic Mallory–Denk inclusion bodies [8]. Biopsy may yield prognostic information; for example neutrophil infiltration correlates positively with CS response [9] and short-term survival [10]. Doppler ultrasonography and scintigraphy with ^99m^Tc-colloids [11,12] may support the diagnosis but lack specificity. There is therefore a recognised need for non-invasive diagnostic tests [3].

The predominating inflammatory cell infiltrating the liver in SAH is the neutrophil [8]. Almost all inflammatory diseases involving neutrophils have been imaged at some time with radiolabelled leucocytes, most notably inflammatory bowel disease in a gastroenterological context [13]. The labelled neutrophil is the effective ingredient in labelled leucocytes, which therefore depict not only neutrophil-predominant inflammation but also physiological neutrophil kinetics, especially neutrophil transit through the lungs [14], pooling (i.e. slow intravascular transit) in tissues (specifically, liver, spleen and bone marrow) [15], and sites of physiological neutrophil destruction (again, liver, spleen and bone marrow) [16]. Despite their widespread use in other scenarios, labelled leucocytes have never previously been used to diagnose hepatic inflammation, probably because the liver is a major site of physiological neutrophil pooling and destruction, and consequently regarded as beyond such inflammation-targeted imaging.

In SAH, however, we speculated that physiological neutrophil pooling and destruction would be sufficiently impaired to expose pathological neutrophil migration in the liver and thereby allow imaging of inflammation with labelled leucocytes or purified neutrophils. This would open up two potentially important applications: firstly, diagnosing SAH non-invasively in the appropriate clinical setting, and secondly providing a platform for testing new anti-inflammatory therapies, analogous to the use of ^111^In-labelled neutrophils and eosinophils in inflammatory lung disease [17,18]. The aim of the current study, therefore, was to investigate the potential of ^111^In-labelled leucocytes to non-invasively image SAH.

## Methods

This was a prospective study involving three groups of patients.

1. Subjects with SAH (*n*=19) who were recruited from consecutive hospital admissions. SAH was suspected on clinical grounds from an admission DF ≥32 in active or recently abstinent heavy alcohol consumers with jaundice for ≤3 months, and confirmed wherever possible by TJB. All patients were screened to exclude other causes of liver disease, coexistent biliary obstruction and focal hepatic lesions. Medical therapy for SAH with CS (prednisolone 40 mg daily) and/or pentoxifylline (PTX) (1200 mg daily) was prescribed at the discretion of the managing hepatologist. The response to treatment was determined by a fall in bilirubin level following a week of therapy [19]. Two patients received no specific treatment for SAH and three were given blinded therapy as part of another placebo-controlled study [20], preventing assessment of treatment response. Therapy was started after imaging in five patients, while in the remaining nine the median duration of treatment at the time of scintigraphy was 2 (1.5–6.5) days.
2. Patients with alcohol-related cirrhosis but without SAH (*n*=14) who were recruited following abstinence from alcohol for ≥6 months to ensure resolution of subclinical steatohepatitis. Cirrhosis was diagnosed from the combination of cutaneous stigmata of chronic liver disease, radiological findings (irregular liver margin with or without splenomegaly) and corroborative histology, where available.
3. Controls without liver disease (*n*=11) who were referred for ^111^In-leucocyte scintigraphy for suspected prosthetic joint infection. They had normal liver biochemistry, no risk factors for liver disease and no evidence of inflammatory pathology on ^111^In-leucocyte scintigraphy, so could be regarded as normal controls. Those reporting alcohol use exceeding Royal College of Physicians guidelines on safe consumption were excluded. The study received ethical approval via the NHS National Research Ethics Service (reference number 08/H1107/36).

### Leucocyte labelling

Autologous leucocytes from peripheral venous blood were labelled *in vitro* under sterile conditions with the lipophilic chelating agents, ^111^In-oxine (*n*=41) or ^111^In-tropolonate (*n*=3, all with SAH) according to published guidelines [21]. Briefly, erythrocytes were allowed to sediment from 35-ml anticoagulated blood, aided by the addition of 1% methylcellulose. A leucocyte-rich, platelet-depleted cell pellet was obtained by centrifugation of the supernatant. The cells were re-suspended in saline (^111^In-oxine) or plasma (^111^In-tropolonate) and incubated with ~25 MBq of ^111^In-chelate for 15 min, before addition of autologous platelet-poor plasma. The labelled leucocytes were pelleted, supernatant aspirated, and cell-associated and unbound radioactivity measured to calculate labelling efficiency. ^111^In-labelled leucocytes were re-suspended in a further 3 ml of platelet-poor plasma and injected intravenously. The administered radioactivity was ~20 MBq, giving a radiation exposure of ~7 mSv.

### ^111^In-leucocyte imaging protocol

Static planar images of the chest and abdomen were obtained 30 min and 24 h after administration of labelled leucocytes using a dual-headed γ camera (SMV DSTXL or GE Discovery NM630) with medium energy collimators and a 10-min acquisition time. To assess labelled cell viability, the recovery of cell-bound label was determined from peripheral venous blood samples obtained 45 min post injection and expressed as a percentage of administered activity.

### ^111^In-leucocyte image analysis

Tissue-associated radioactivity (expressed as mean counts per pixel) was determined from manually defined regions of interest (ROI) over homogeneous areas of liver, spleen and lungs. The geometric mean of anterior and posterior counts was corrected for physical radionuclide decay and background activity. The change in liver-associated radioactivity between the two imaging times was expressed as the 24 h:30 min ratio of activities.

### ^99m^Tc-nanocolloid scintigraphy

^99m^Tc-nanocolloid scintigraphy was performed after completion of ^111^In-leucocyte scintigraphy in 11 patients with SAH and 7 abstinent patients with cirrhosis. Abdominal γ camera images (SMV DSTXL) were acquired 20 min post injection of 80 MBq ^99m^Tc-nanocolloid (Nanocoll, GE Healthcare, Amersham, U.K.; radiation exposure ~1 mSv) using low-energy high-resolution collimation. Down-scatter from the ^111^In photopeak accounted for <15% of ^99m^Tc tissue activity. Geometric means of counts per pixel were calculated for liver and spleen in the same ROI as above and expressed as liver:spleen ratio.

### Liver biopsy

TJB (radiation exposure ~10 mSv) was performed in patients with SAH within 1 week of ^111^In-leucocyte scintigraphy. Independent biopsy interpretation was undertaken by two histopathologists blinded to the other’s findings and to scintigraphy. SAH was defined as the coexistence of steatosis, hepatocyte ballooning and lobular neutrophil infiltration [3]. Histological features of steatohepatitis, including hepatocyte ballooning, lobular inflammation, steatosis, canalicular cholestasis, ductular cholestasis and cholangiolitis, were assessed according to recently described criteria [4]. The severity of steatohepatitis was semiquantificatively classified as mild, moderate or marked according to whether foci of infiltrating parenchymal neutrophils were sparse, moderate or abundant. Cells positive for granulocyte marker CD15 were counted in five to ten non-consecutive high-power fields in 13 patients in whom sufficient biopsy material was available for immunohistochemical staining, and the median count averaged between the two histopathologists.

### Microautoradiography

To determine the intrahepatic fate of ^111^In-leucocytes in SAH, microautoradiography was performed on biopsy specimens obtained 24 h post-injection of labelled leucocytes in a single patient. Formalin-fixed liver sections (3 μm thickness) were placed on positively charged adhesive slides, de-waxed and a radiosensitive emulsion (Ilford K2, Harman Technology, U.K.) applied in dark room conditions. Microautoradiographs were exposed in dry, dark conditions for 2 weeks, following which they were fixed, developed, dried, post stained with H&E and inspected for foci of radioactivity.

### Statistical analysis

Data are presented as mean ± S.D. or median (interquartile range). Parametric data were compared using Student’s *t*test or ANOVA and non-parametric data using Mann–Whitney *U* or Kruskal–Wallis tests. Correlation was quantified using Pearson’s correlation coefficient (*r*) or Spearman’s rho (ρ) for parametric and non-parametric data respectively. Interobserver agreement of categorical histopathology data was assessed using the κ statistic (κ). Reported *P*-values are two-tailed.

## Results

### Patients

Baseline characteristics and demographic data are shown in Table 1. Median alcohol consumption prior to admission was 144 g/day (80–200) for 10 years [5–15]. Four abstinent cirrhotics had histologically confirmed disease. The diagnosis in the remaining seven rested on clinical and radiological grounds.

**Table 1:** Demographics and patient characteristics at recruitment

	SAH (*n*=19)	Abstinent cirrhosis (*n*=14)	Normal controls (*n*=11)	*P*
Age (years)	50.3 ± 9.8	55.7 ± 8.9	64.0 ± 14.9*	0.009
Male gender	14 (73.7%)	11 (78.6%)	7 (63.6%)	0.702
Ascites	11 (57.9%)*	2 (14.3%)	-	0.015
SIRS	4 (21.1%)	-	-	
Child-Pugh score	10 (8–11)*	5 (5–6)	-	<0.0001
MELD score	23 (22–27)*	8.8 (7.4–9.3)	-	<0.0001
DF	52 (44–75)	-	-	-
GAHS	9 (8–10)	-	-	-
GAHS ≥ 9	12 (63.2%)	-	-	-
Leucocyte count (× 10^9^/l)	12.6 (9.3–15.9)*	6.6 (5.6–7.6)	7.0 (6.3–8.7)	<0.001
Neutrophil count (× 10^9^/l)	9.8 (7.1–13.3)*	3.9 (2.6–4.9)	4.3 (3.7–5.5)	<0.001
Platelet count (× 10^9^/l)	158 (106–264)	136 (91–194)*	239 (174–287)	0.047
INR	1.8 (1.6–2.1)*	1.1 (1.1–1.2)	1.1 (1.0–1.1)	<0.0001
Bilirubin (μmol/l)	288 (202–451)*	10 (8–17)	7 (4–11)	<0.0001
Albumin (g/l)	29 (27–34)*	43.5 (37–46)	43 (42–44)	<0.0001
Creatinine (μmol/l)	56 (47–102)	80.5 (62–107)	78 (58–81)	0.085
ALT (U/l)	63 (49–80)*	17.5 (15–32)	20 (15–22)	<0.0001
AST (U/l)	171 (117–220)*	36.5 (25–46)	18 (14–20)	<0.0001

Data are presented as mean ± S.D., median (IQR) or number (%). Abbreviations: ALT, alanine aminotransferase; AST, aspartate aminotransferase; GAHS, Glasgow alcoholic hepatitis score; INR, international normalised ratio; IQR, interquartile range; MELD, modified end-stage liver disease score; SIRS, more than or equal to two features of the systemic inflammatory response syndrome at recruitment.

Normal values: bilirubin 0–21 μmol/l, INR 0.8–1.2, albumin 35–52 g/l, ALT 0–41 iu/l, AST 0–40 iu/l, creatinine 62–106 μmol/l, leucocyte count 4–11 × 10^9^/l, neutrophil count 2–7.5 × 10^9^/l, platelet count 150–450 10^9^/l. *P<0.05.

In patients with SAH, the median admission DF was 52 (44–75); the Glasgow Alcoholic Hepatitis Score (GAHS) [22] was ≥9 in 12 and modified end-stage liver disease (MELD) [23] score ≥21 in 16. Four (21%) had evidence of the systemic inflammatory response syndrome (SIRS) and two had active infection (limb cellulitis and spontaneous bacterial peritonitis (SBP)). Peripheral blood leucocyte counts were significantly higher in SAH than in either of the other groups (Table 1; *P*<0.001). Normal controls were significantly older than either cohort with liver disease (*P*=0.009), consistent with the population referred for investigation of prosthetic joint pain.

### ^111^In-labelled leucocyte scintigraphy

**Figure 1:**
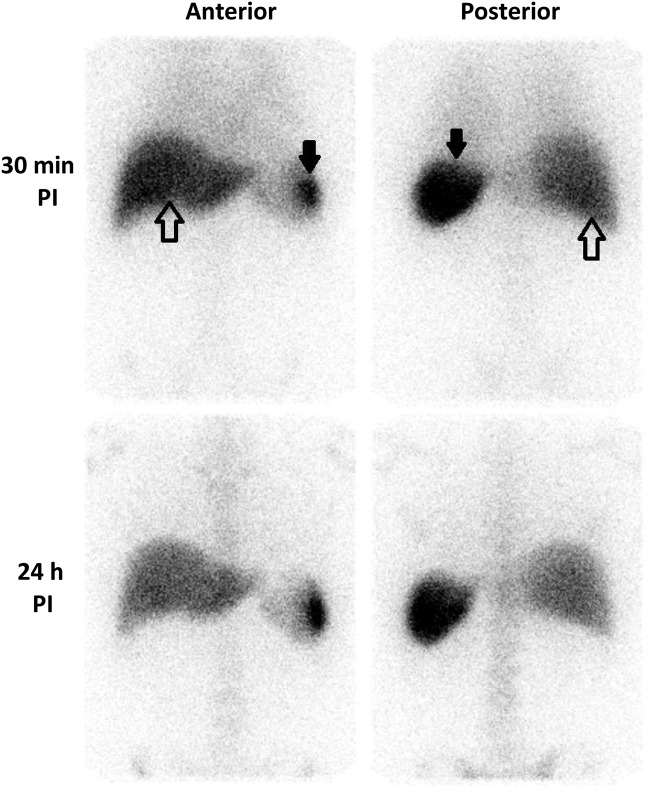
Physiological ^111^In-labelled leucocyte distribution γ camera images (anterior and posterior projections) obtained 30 min and 24 h post injection of autologous ^111^In-labelled leucocytes in a normal control. Liver: open arrows; spleen: closed arrows. Hepatic and splenic activities reflect physiological leucocyte pooling at 30 min and cell destruction at 24 h. Hepatic activity remains unchanged or decreases between the two time points. Lung activity can be seen faintly at 30 min.

Labelling efficiency was broadly similar among the three patient groups: 89 (85–92)%, 84 (81–85)% and 86 (81–88)%. Labelled cell recovery, however, was significantly higher in SAH (57 (48–63)%) compared with abstinent cirrhotics and normal controls (38 (26–46)% and 38 (34–44)%, respectively, *P*=0.002),

Normal ^111^In-leucocyte scintigraphy 30-min post injection shows prominent activity in liver and spleen, and faint diffuse activity in the lungs. At 24 h, the diffuse lung activity disappears and bone marrow activity becomes evident (Figure 1). Liver activity falls slightly while spleen remains largely unchanged, suggesting that leucocyte pooling is broadly matched by neutrophil destruction [13].

**Figure 2:**
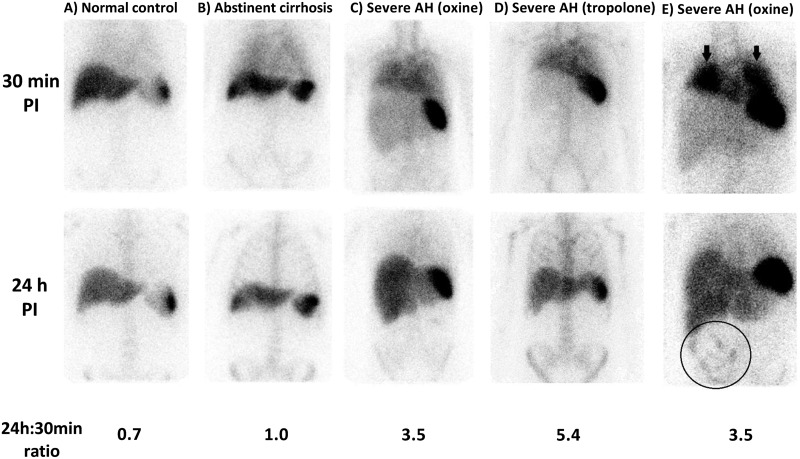
Examples of ^111^In-leucocyte scintigraphy appearances in SAH compared with normal and cirrhotic controls Example anterior γ camera images following ^111^In-leucocyte administration with corresponding 24 h:30 min hepatic activity ratios in normal control (**A**), abstinent control with compensated alcohol-related cirrhosis (**B**), patients with SAH studied using ^111^In-oxine labelling (**C**) and ^111^In-tropolonate labelling (**D**). Extrahepatic findings in SAH included prominent lung activity at 30 min post-injection (arrows) and gut activity at 24 h (circled) (**E**).

**Figure 3:**
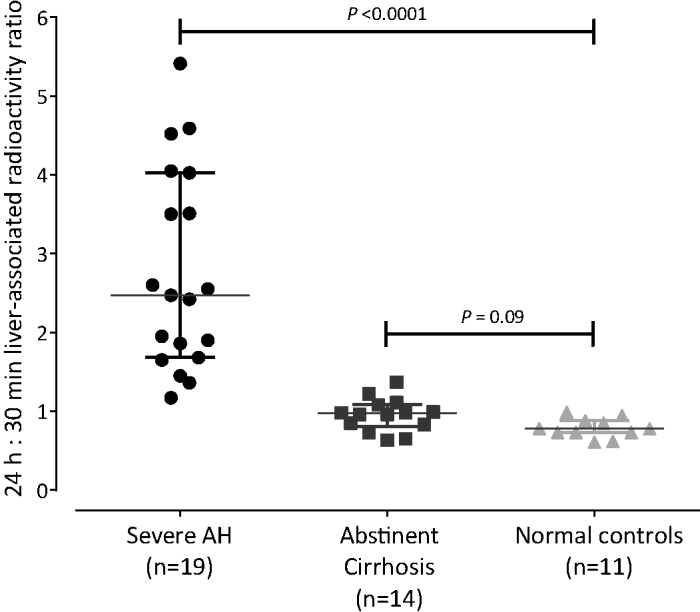
24 hour : 30 minute liver activity ratios at ^111^In-leucocyte scintigraphy in SAH, compensated cirrhosis and normal controls The 24 h:30 min ratios were significantly higher in severe AH than controls with inactive cirrhosis and normal controls. Bars depict median value, whiskers IQR (interquartile range).

The 24 h:30 min activity ratio in the liver was significantly higher in SAH (2.5 (1.7–4.0) compared with abstinent cirrhotics (1.0 (0.8–1.1)) and controls (0.8 (0.7–0.9), *P*<0.0001; Figures 2 and 3). Scintigraphic appearances in SAH were similar between ^111^In-oxine and ^111^In-tropolone radiolabelling (Figure 2). Two abstinent cirrhotics had decompensated liver disease with diuretic-refractory ascites and liver synthetic impairment (both Child–Pugh class B). The 24 h:30 min ratio in these two individuals (1.08 and 1.37) was not significantly higher than in compensated cirrhosis (median 0.96 (0.78–0.99), *P*=0.067) and remained substantially lower than the cohort with SAH (*P*=0.025).

Prominent diffuse pulmonary activity at 30 min, which cleared by 24 h, was a frequent finding in SAH (Figure 2) and occurred to a lesser degree in abstinent cirrhotics. Median lung-associated radioactivity at 30 min (expressed relative to splenic activity) was significantly higher in SAH (20 (15–26)%) than normal controls (14.5 (13–16)%; *P*=0.02), but did not differ significantly from abstinent cirrhotics (18 (12.5–25)%, *P*=0.38). By 24 h, lung radioactivity had decreased the 24 h:30 min hepatic activity ratio in patients with SAH, abstinent cirrhotics and normal controls (bars depict median value, whiskers show interquartile range (IQR)). Similarly in all three groups but could not be quantified because of overlying physiological activity in chest wall bone marrow.

Abnormal gut-associated radioactivity 24 h post-injection was observed in four patients with SAH (Figure 2). Delayed imaging at 48–72 h demonstrated distal transit of ^111^In within bowel, in keeping with access of labelled leucocytes into bowel.

### Liver histology in SAH

Clinical deterioration after ^111^In-leucocyte scintigraphy precluded TJB in two patients with SAH, leaving 17 with histology. The majority had underlying advanced hepatic fibrosis or cirrhosis and all exhibited some degree of steatohepatitis, judged to be marked in seven (Table 2). Interobserver agreement of fibrosis stage, steatosis grade, steatohepatitis severity and presence of Mallory’s hyaline was high (κ ≥ 0.5), but there was substantial variability in assessment of ballooning grade, degree of canalicular cholestasis and the presence of cholangiolitis and Councilman bodies (κ ≤ 0.2 in all).

**Table 2:** Summary of dual histopathologist liver biopsy interpretation in SAH (*n*=17)

Histological parameter	Pathologist #1 *n* (%)	Pathologist #2 *n* (%)	Agreement κ (*P*)
**Ishak fibrosis stage**			
Cirrhosis	9 (52.9%)	10 (58.8%)	0.538
Stage 5	7 (41.2%)	2 (11.8%)	(*P*<0.0001)
Stage 4	-	1 (5.9%)	
Stage 3	-	3 (17.6%)	
Stage 2	1 (5.9%)	1 (5.9%)	
**Steatosis (parenchymal involvement)**			
<5%	1 (5.9%)	2 (11.8%)	0.750
5–33%	6 (35.3%)	6 (35.3%)	(*P*<0.0001)
>33–66%	7 (41.2%)	5 (29.4%)	
>66%	3 (17.6%)	4 (23.5%)	
**Severity of lobular neutrophil infiltrate**			
Minimal	2 (11.8%)	3 (17.6%)	0.904
Moderate	8 (47.1%)	7 (41.2%)	(*P*<0.0001)
Marked	7 (41.2%)	7 (41.2%)	
**Parenchymal involvement by ballooning**			
No ballooning	-	-	
<5%	1 (5.9%)	2 (11.8%)	0.183
>5–10%	1 (5.9%)	4 (23.5%)	(*P*=0.160)
>10–20%	6 (35.3%)	3 (17.6%)	
>20–50%	8 (47.1%)	7 (41.2%)	
>50%	1 (5.9%)	1 (5.9%)	
**Canalicular cholestasis number of bile plugs in canaliculi**			
None	5 (29.4%)	5 (29.4%)	0.174
<5	-	9 (52.9%)	(*P*=0.033)
>5–10	1 (5.9%)	1 (5.9%)	
>10–20	8 (47.1%)	2 (11.8%)	
>20	3 (17.6%)	-	
**Ductular cholestasis number of bile plus in ductules**			
None	14 (82.4%)	14 (82.4%)	0.218
1	2 (11.8%)	3 (17.6%)	(*P*=0.285)
2	-	-	
3	-	-	
>3	1 (5.9%)	-	
**Mallory’s hyaline**	14 (82.4%)	15 (88.2%)	0.767
			(*P*=0.001)
**Cholangiolitis**	13 (76.5%)	12 (70.6%)	–0.053
**Polymorphs around and in ductule lumen**			(*P*=0.825)
**Councilman bodies**	9 (47.1%)	4 (23.5%)	0.029
			(*P*=0.893)

**Figure 4:**
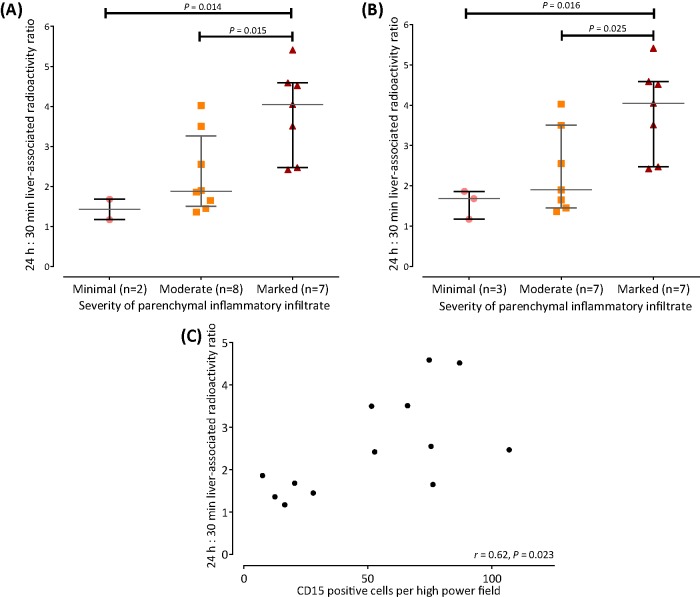
Comparison between ^111^In-leucocyte scintigraphy findings and liver histology in SAH The 24 h:30 min hepatic activity ratios were significantly higher in marked compared with mild and moderate steatohepatitis (pathologist #1 (**A**) and pathologist #2 (**B**)). CD15 quantification of parenchymal neutrophil infiltration correlated with 24 h:30 min activity ratio (**C**).

There was an agreement between histopathologists regarding severity of parenchymal neutrophil infiltration in 16 out of 17 patients. Steatohepatitis was deemed marked in seven patients, moderate in seven and mild in two. The 24 h:30 min hepatic activity ratio was significantly higher in marked steatohepatitis (4.0 (3.0–4.6)) compared with moderate (1.9 (1.6–3.0)) and mild (1.45 (1.2–1.7)) steatohepatitis (*P*=0.022; Figure 4). The 24 h:30 min ratio in patients with histologically mild steatohepatitis was similar to that in abstinent cirrhotics with hepatic decompensation (*P*=0.25).

There was good interobserver agreement in parenchymal granulocyte quantification determined from CD15-positive cell counts *(r* = 0.85, *P*<0.001*).* CD15-positivity count correlated modestly with the 24 h:30 min ratio (Figure 4; *r* = 0.62; *P*=0.023).

**Figure 5:**
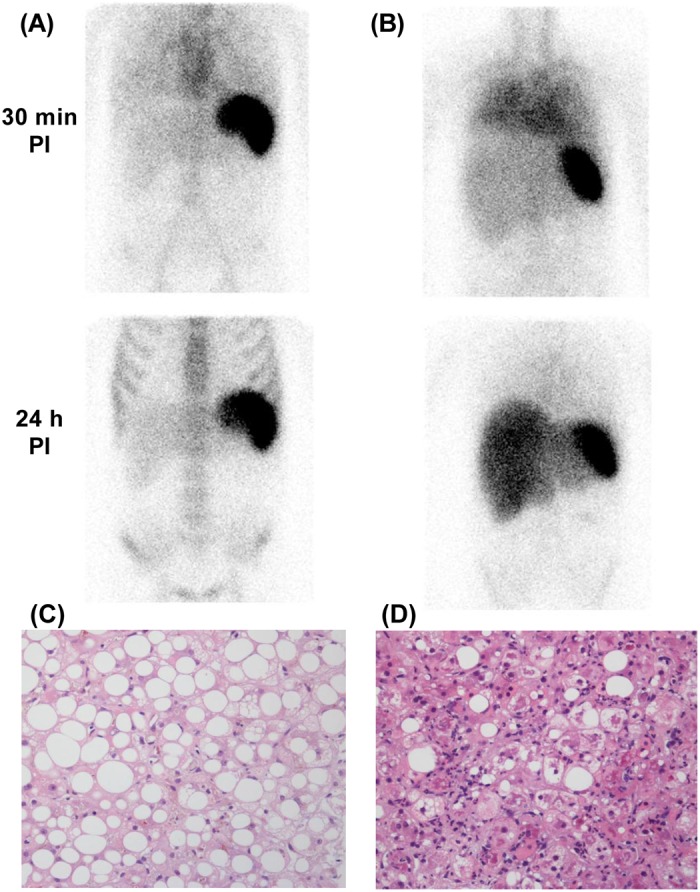
Example ^111^In-leucocyte scintigraphy and corresponding liver histology in SAH patients with mild and marked steatohepatitis ^111^In-leucocyte scintigraphy in patients with mild (**A**) and marked (**B**) steatohepatitis with corresponding Haematoxylin and Eosin (H&E) stained liver biopsy sections (**C**,**D** respectively). Note increased diffuse lung activity at 30 min post injection and hepatomegaly, plus a hint of gut activity in the left mid-abdomen, in (**B**). The 24 h:30 min hepatic activity ratios were 1.2 and 3.5 respectively. Note that in (**B**) lung activity clears by 24 hours.

Examples of ^111^In-leucocyte scintigraphy and corresponding liver biopsy sections in mild compared with marked steatohepatitis are shown in Figure 5. No association was observed between the 24 h:30 min activity ratio and fibrosis stage.

### Clinical parameters, treatment response and survival

There was no correlation between the 24 h:30 min activity ratio and serum bilirubin (*ρ* = 0.02, *P*=0.9), peripheral blood leucocyte counts (*ρ* = 0.28, *P*=0.25) or clinical measures of disease severity, including Child–Pugh score (*ρ* = –0.17, *P*=0.5), DF (*ρ* = 0.16, *P*=0.5), GAHS (*ρ* = 0.29, *P*=0.23) or MELD (*ρ* = 0.26, *P*=0.29). Assessment of treatment response in SAH was precluded in three patients who were treated as part of another placebo-controlled trial. Of the remaining 16, 14 received CS and/or PTX, of whom 10 exhibited a clinical response following a week of therapy. No significant difference was observed in the 24 h:30 min activity ratio between patients who responded to therapy compared with non-responders (2.4 (1.6–3.8) compared with 2.3 (1.7–2.6)). Inpatient mortality (21%) was not predicted by imaging findings. Furthermore, there was no association between histological findings and either treatment response or in-hospital survival.

### Microautoradiography

**Figure 6:**
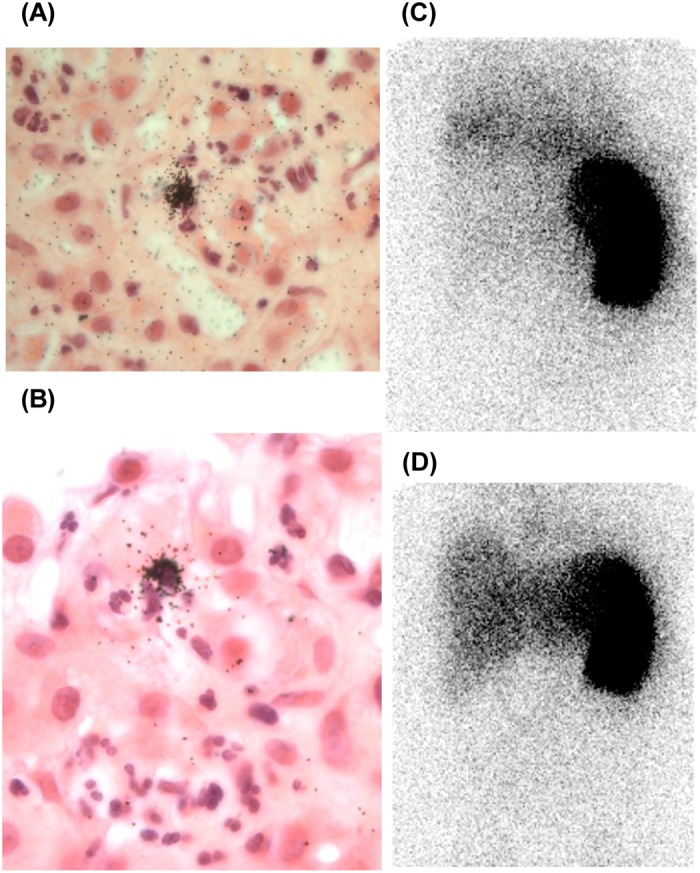
Liver biopsy microautoradiography in SAH Liver microautoradiographs in SAH from biopsy material obtained 24h post injection (PI) of ^111^In-labelled leucocytes. H&E stained microautoradiographs at 40× (**A**) and 100× (**B**) objective magnification demonstrate foci of parenchymal granulocyte-associated radioactivity, indicated by overlying clusters of black silver halide crystals, against a low level of background radioactivity. Corresponding γ camera images 30 min (**C**) and 24 h (**D**) PI demonstrate a prominent increase in liver-associated radioactivity (24 h:30 min activity ratio of 4.5). Note that in (D) lung activity clears by 24h.

H&E-stained autoradiography undertaken in one patient with SAH demonstrated foci of cell-associated radioactivity in areas of parenchymal neutrophil infiltration with low level background hepatic radioactivity (Figure 6). The 24 h:30 min hepatic activity ratio in this patient was 4.5.

### ^99m^Tc-nanocolloid scintigraphy

**Figure 7:**
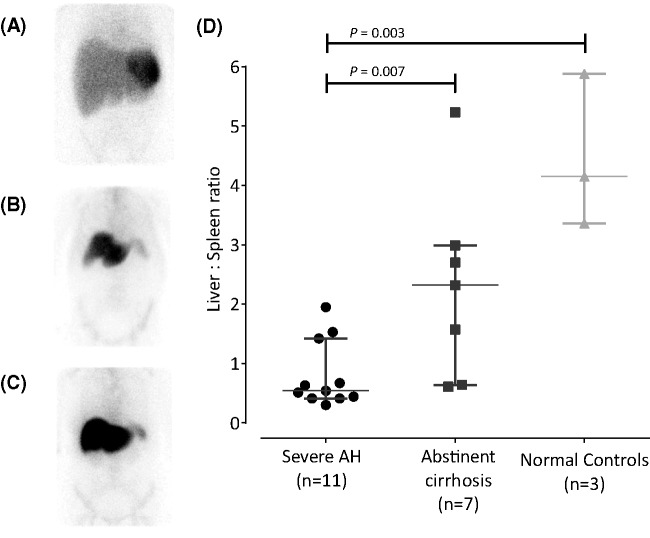
Technetium-99m (^99m^Tc) nanocolloid scintigraphy Example anterior γ camera images following^ 99m^Tc nanocolloid administration in severe AH (**A**), demonstrating liver ‘drop out’ compared with inactive cirrhotic (**B**) and normal controls (**C**). Hepatic:splenic ^99m^Tc-nanocolloid activity ratios (geometric mean of counts/pixel) were significantly lower in SAH compared with both abstinent cirrhotics and normal controls, consistent with severely impaired liver reticuloendothelial function (**D**).

Hepatic-to-splenic uptake ratio of ^99m^Tc-nanocolloid was significantly lower in SAH (median 0.5 (0.4–1.4)) compared with abstinent cirrhotics (2.3 (0.6–3.0)) and three historic controls without liver disease (4.2 (3.8–5.0); *P*=0.003; Figure 7). In SAH patients, however, there was no correlation between ^99m^Tc-nanocolloid liver/spleen ratios and either the 24 h:30 min activity ratio (*r* = −0.04, *P*=0.9) or histological severity of steatohepatitis (*r* = −0.4, *P*=0.3).

## Discussion

This proof-of-concept study shows for the first time that hepatic neutrophil migration can be successfully imaged and semiquantified using ^111^In-leucocyte scintigraphy. This provides not only a potential means of diagnosis and outcome prediction in SAH, but also, importantly, a platform for the assessment of new anti-inflammatory therapies for a range of acute inflammatory liver disorders. An analogous scenario is inflammatory pulmonary disease in which there is interest in using labelled neutrophils and eosinophils for assessing new therapies. While labelled ‘mixed’ leucocytes are shown here to be appropriate for routine clinical use, labelled purified neutrophils would probably be preferable for research applications.

Current European guidelines recognise the need for non-invasive diagnostic tools for SAH [3]. Abnormal leucocyte scintigraphy in SAH was characterised by an increase in liver-associated radioactivity between 30 min and 24 h, not seen in abstinent cirrhotics or normal controls. The 24 h:30 min hepatic activity ratio portrays intrahepatic neutrophil migration as shown by correlation with measures of neutrophil infiltration on liver biopsy.

We hypothesised that, for whatever mechanistic reason but almost certainly including reduced hepatic blood flow, physiological hepatic accumulation of ^111^In, both at 30 min as a result of impaired pooling, and 24 h as a result of impaired destruction, would be sufficiently decreased in SAH to expose hepatic neutrophil migration as an increase in liver-associated radioactivity between 30 min and 24 h. Consistent with impaired neutrophil destruction, hepatic accumulation of ^99m^Tc-nanocolloid was suppressed in SAH (Figure 7), corroborating the findings of earlier studies indicating impaired Kupffer cell phagocytic function [12,24]. The increase in liver-associated ^111^In activity is almost certainly, therefore, the result of neutrophil migration. This interpretation is supported by microautoradiography of biopsy material obtained 24 h post-injection of ^111^In-leucocytes, which demonstrated, albeit in only one individual, discrete foci of cell-associated radioactivity in areas of neutrophil satellitosis within the lobular parenchyma. Moreover, although it provides no insight into the mechanisms of cellular extravasation, CD15 expression correlated significantly with the 24 h:30 min activity ratio.

Several extrahepatic features were noted on ^111^In-leucocyte scintigraphy. Prominent diffuse lung radioactivity at 30 min, clearing by 24 h, was a consistent finding in SAH, suggesting pulmonary vascular entrapment of primed neutrophils without extravascular migration. These imaging features have been reported in several other disorders associated with systemic neutrophil priming, including vasculitis [25] and severe inflammatory bowel disease [26]. Leucocyte kinetics associated with priming are different from those seen following labelling-induced leucocyte injury, which gives immediate lung activity, markedly reduced 45 min recovery and decreased physiological uptake in the spleen and bone marrow. Our scintigraphic findings of priming add to the existing *ex vivo* evidence for systemic neutrophil priming in SAH [27] and suggest that priming also occurs, albeit to a lesser degree, in patients with compensated chronic liver disease.

Labelled leucocyte recovery is probably the best benchmark of cell ‘health’ following labelling. The marginated neutrophil pool (which in health resides in the liver, spleen and bone marrow) [16] is in equilibrium with the circulating pool, and represents ~50% of the total blood neutrophil pool. Maximum recovery is therefore ~50% of administered activity. The increased recovery in SAH, despite increased pulmonary margination, suggests a pathophysiological shift in granulocyte margination away from the liver, in keeping with clearly reduced hepatic activity at 30 min post injection in SAH (Figure 2). On the other hand, delayed pulmonary transit would be expected to reduce recovery.

Abnormal gut radioactivity was observed in a fifth of patients with SAH, in the absence of gastrointestinal (GI) symptoms or apparent disease. Because ‘free’ ^111^In does not enter the gut, distal transit of ^111^In on delayed imaging suggests luminal transit of labelled cells within faeces. The cause remains unclear and warrants further study, although potential explanations include disposal of intrahepatic neutrophils cells via bile, or more likely a sequel of portal hypertension.

We did not identify patients with clinically suspected SAH where histology refuted or altered the diagnosis, as others have shown [4,6]. Our data, however, demonstrated significant heterogeneity in the severity of steatohepatitis that was independent of clinical and laboratory disease markers, but predicted by ^111^In-leucocyte scintigraphy. No correlation was observed between clinical prognostic scores commonly used to judge disease severity and either the 24:30 min activity ratio or histological severity of steatohepatitis. However, most patients had clinically advanced disease making it difficult to further stratify according to disease severity.

Although recent clinical trials have questioned the benefit from CS [20], they remain widely used in medical management of SAH. Previous authors described a positive association between parenchymal neutrophil infiltration and clinical CS response in SAH [9]. Predicting response to therapy would be useful, particularly given the high rates of infection and poor survival in CS non-responders [28]. We did not demonstrate a relationship between scintigraphic findings and either treatment response or in-hospital mortality. Furthermore, we saw no association between steatohepatitis severity and either treatment response or survival, probably because of heterogeneity in prescribed therapies and small patient numbers. A larger study, powered to assess these specific end points, would be required to assess the prognostic value of ^111^In-leucocyte scintigraphy.

Further study limitations are as follows. For the convenience that would be important in routine clinical studies, we used labelled ‘mixed’ leucocytes. However, although mixed leucocytes clearly demonstrate the feasibility of imaging hepatic inflammation, ^111^In-labelled purified neutrophils would be preferable for research applications. ^111^In is preferable to ^99m^Tc because of its greater cell labelling stability. Moreover, ^111^In preferentially labels neutrophils, unlike ^99m^Tc-hexamethylpropylamine oxime (HMPAO), which is strongly selective for eosinophils [29].

Differentiation of SAH from other acute deteriorations of chronic liver disease, particularly end-stage cirrhosis or decompensation due to infection, is often challenging and any non-invasive diagnostic test for SAH would need to distinguish it from decompensation. Although they will form the cohort for future studies, we did not include a control group with decompensated cirrhosis at this stage of our work as our primary aim was to establish the feasibility of imaging hepatic inflammation with radiolabelled leucocytes in homogeneous patient cohorts. A decompensated group would be highly heterogeneous and likely to include patients with ongoing or recent alcohol use, in whom subclinical AH would be a significant unmeasured confounder. Confident inclusion of such patients, moreover, would have required TJB for the sole purpose of excluding those with histologically active steatohepatitis, and would therefore have raised significant ethical issues. TJB in SAH, in contrast, is clinically justifiable and indeed standard of care in some centres. Liver biopsy is not common practice in other forms of ARLD, which are invariably associated with ongoing alcohol use. Given their high prevalence of asymptomatic subclinical steatohepatitis, active heavy drinkers [30], other than SAH patients, were not included. Patients with cirrhosis were therefore recruited following at least 6 months abstinence, to allow sufficient time for resolution of underlying steatohepatitis.

In conclusion, we have demonstrated how ^111^In-labelled leucocyte scintigraphy can be exploited to detect hepatic neutrophil migration in SAH. The technique has potential clinical application as a non-invasive means of diagnosing this condition and assessing the severity of liver inflammation without the need for liver biopsy. Importantly, it also has the potential to provide a platform for experimental therapies aiming to inhibit intrahepatic neutrophil migration. Evaluation of leucocyte kinetics in other forms of acute and acute-on-chronic liver disease would be of interest.

## Abbreviations

AH: alcoholic hepatitis
ARLD: alcohol-related liver disease
CS: corticosteroids
DF: discriminant function
GAHS: Glasgow alcoholic hepatitis score
MELD: modified end-stage liver disease
PTX: pentoxifylline
SAH: severe AH
SIRS: systemic inflammatory response syndrome
